# Bibliographical Notices

**Published:** 1847-07

**Authors:** 


### BIBLIOGRAPHICAL NOTICES.

*Catalogue of Anatomical Preparations.* By J. C. & D. Hyatt. New York City. 1847.

"*Itaque, ista naturae rerum contemplatio, quamvis non faciat medicum, aptiorem tamen medicinae reddit.*"—Com. Cels., Praefat.

This is a volume of no mean dimensions, wholly occupied by a synoptical description of the various preparations manufactured by those gentlemen. The materials of which the articles are made, is a secret which belongs only to the inventors, and from which they are in a fair way of securing a golden harvest. Among many other things, we notice the "*Complete Model of a Man,* which can be separated into 130 parts, and showing 1,700 objects in detail"—price $250. A "*complete larynx,* much magnfied, and showing all its anatomy in detail"—price $5,00. The eye, magnified ;—deformed female pelvis ;—seven periods of utero gestation, &c., &c. They also keep for sale, carefully prepared, skeletons, pathological specimens, &c., Such an establishment has long been needed in this country ; and we congratulate the profession that hereafter they will not be compelled to depend upon foreign countries to furnish their cabinets. F. H. H.

---

*The Pathological Anatomy of the Human Body.* By Julius Vogel, M. D., Professor of Clinical Medicine at the University of Giessen. Translated from the German, with additions, by Geo. E. Day, M. A., and L. M. Cantab, &c. Illustrated by upwards of one hundred plain engravings. Philadelphia. Lea & Blanchard. 1847. 8vo. pp. 534.

This is a treatise on *general* morbid anatomy, comprising the results of chemical and microscopical investigations, which, within a few years past, have given a new aspect and character to this fundamental branch of philosophical and practical medicine. It is a valuable addition to medical literature, and is entitled to be considered a standard authority on the subject of which it treats. Our present purpose is to present to our readers, in a future number, an analytical notice of some portions of this work. The author promises another treatise, to be devoted to the pathological changes affecting special organs.

---

*A System of Human Anatomy, General and Special.* By Erasmus Wilson, M. D., Lecturer on Anatomy, London. Third American, from the Third London Edition. Edited by Paul B. Goddard, A. M., M. D., Professor of Anatomy, &c., &c., in the Franklin Medical College, of Philadelphia: with two hundred and thirty-three Illustrations by Gilbert. Philadelphia. Lea & Blanchard. 1847. 8vo. pp. 610.

The high estimation in which this work is held by the profession, has already created the necessity of a third edition, both in this country and Great Britain, although it is but a few years since the first edition was issued. We believe it is recommended in most of our medical colleges, as one of the best of text books for the anatomical student. It embraces a summary of the recent developments in histology, and, in all respects, appears to represent the present advanced condition of the science of anatomy.

---

*A Treatise on the Diseases of the Eye.* By W. Lawrence, F.R.S., Surgeon Extraordinary to the Queen, &c. A new edition, edited, with numerous additions, and one hundred and seventy-six illustrations, by Isaac Hays, M. D., Surgeon to Wills' Hospital, &c. Philadelphia. Lea & Blanchard. 1847. 8vo. pp. 859.

The original work, by Lawrence, as a comprehensive, accurate, and practical treatise on ophthalmic science, is only equalled by that of M'Kenzie, of Glasgow. As presented, however, to the American student and practitioner, with the copious and valuable additions by Dr. Hays, it certainly has no superior. All who are desirous of availing themselves of the most modern and approved principles in this interesting and important province of surgical science, should not fail to possess the latest edition of this sterling work.

